# Early initiation of renal replacement therapy in critically ill patients: a meta-analysis of randomized clinical trials

**DOI:** 10.1186/s12871-019-0733-7

**Published:** 2019-05-01

**Authors:** Laura Pasin, Sabrina Boraso, Ivo Tiberio

**Affiliations:** Department of Anesthesia and Intensive Care, Ospedale S. Antonio, Via Facciolati, 71 Padova, Italy

**Keywords:** Renal replacement therapy, Acute kidney injury, Mortality, Intensive care unit

## Abstract

**Background:**

Acute kidney injury (AKI) is strongly associated with high morbidity and mortality of critically ill patients. In the last years several different biological markers with higher sensitivity and specificity for the occurrence of renal impairment have been developed in order to promptly recognize and treat AKI. Nonetheless, their potential role in improving patients’ outcome remains unclear since the effectiveness of an “earlier” initiation of renal replacement therapy (RRT) is still debated. Since one large, high-quality randomized clinical trial has been recently pubblished, we decided to perform a meta-analysis of all the RCTs ever performed on “earlier” initiation of RRT versus standard RRT in critically ill patients with AKI to evaluate its effect on major outcomes.

**Methods:**

Pertinent studies were independently searched in BioMedCentral, PubMed, Embase, and Cochrane Central Register of clinical trials. The following inclusion criteria were used: random allocation to treatment (“earlier” initiation of RRT versus later/standard initiation); critically ill patients.

**Results:**

Ten trials randomizing 2214 patients, 1073 to earlier initiation of RRT and 1141 to later initiation were included. No difference in mortality (43.3% (465 of 1073) for those receiving early RRT and 40.8% (466 of 1141) for controls, *p* = 0.97) and survival without dependence on RRT (3.6% (34 of 931) for those receiving early RRT and 4.2% (40 of 939) for controls, *p* = 0.51) were observed in the overall population. On the contrary, early initiation of RRT was associated with a significant reduction in hospital length of stay. No differences in occurrence of adverse events were observed.

**Conclusions:**

Our study suggests that early initiation of RRT in critically ill patients with AKI does not provide a clinically relevant advantage when compared with standard/late initiation.

**Electronic supplementary material:**

The online version of this article (10.1186/s12871-019-0733-7) contains supplementary material, which is available to authorized users.

## Background

Acute kidney injury (AKI) is a major issue in the intensive care unit (ICU) and is strongly associated with high morbidity and mortality. In fact, despite its potential to be reversed, several studies performed in different clinical settings confirmed that occurrence of AKI is independently associated with in-hospital mortality and negative short- and long-term outcomes of critically ill patients [1–3]. Moreover, early development of AKI during the ICU stay has been shown to be associated with a reduced probability of being alive or having good Health-related quality of life (HRQoL) one year after ICU admission [4].

Given the possible severe implications of this condition, in the last few decades researchers mainly focused their attention on the pathogenesis of AKI and on its prompt recognition, leading to the development of a series of different biological markers with higher sensitivity and specificity for the occurrence of renal impairment [5, 6]. These markers play a fundamental role in the early diagnosis and treatment of AKI [7]. Nonetheless, their potential role in improving patients’ outcome is still debated [8].

In fact, actual indications for renal replacement therapy (RRT) in the ICU require the development of severe clinical manifestations of renal impairment, such as volume overload unresponsive to medical therapy, hyperkaliemia or major electrolyte disturbances, acidosis or uremic complications such as pericarditis or encephalopathy. Whether an “earlier” initiation of RRT might be effective in improving survival of critical ill patients affected by AKI is still debated. Unfortunately, previous meta-analyses on this topic were unconclusive due to the paucity of pubblished data [9–11]. Recently, a large, high-quality randomized clinical trial (RCT), the IDEAL-ICU trial was published in NEJM [12]. In this multicenter trial, 488 adults with septic shock and severe AKI were randomized to receive RRT within 12 h (early strategy) of documented failure stage or after a delay of 48 h (late strategy). Nearly all patients in the early-strategy group received RRT while approximately 30% of patients in the delayed-strategy group did not receive RRT since they had spontaneous recovery of renal function. The IDEAL-ICU trial was stopped early for futility. The primary outcome of mortality at 90 days did not differ between patients who received early versus late initiation of RRT (58% vs. 54%; *P* = 0.38). Furthermore, no benefits were seen from early initiation of RRT in secondary outcomes. Results of previous larger studies were conflicting, Infact, one major trial (ELAIN) showed a 90-day mortality benefit, while another (AKIKI) did not show a benefit at 60 days. The ELAIN trial was smaller, conducted almost exclusively in postoperative AKI patients, and the difference in timing between early versus late initiation of RRT was less than 24 h. Therefore, we decided to perform an updated meta-analysis of all the RCTs ever performed on “earlier” initiation of RRT versus standard RRT in critically ill patients to evaluate its effect on outcome of critically ill patients with AKI.

## Methods

### Search strategy

Pertinent studies were independently searched in BioMedCentral, PubMed, Embase, and the Cochrane Central Register of clinical trials by three investigators. The full PubMed search strategy aimed to include any RCTs ever performed on “earlier” initiation of RRT in critically ill patients with AKI. In addition, we employed backward snowballing (i.e., scanning of references of retrieved articles and pertinent reviews) and contacted international experts for further studies with no language restriction.

### Study selection

The following inclusion criteria were used for potentially relevant studies: studies performed on critically ill patients; random allocation to treatment (“earlier” initiation of RRT versus later/standard initiation). The exclusion criteria were non-adult patients, duplicate publications and lack of data on all of the following: mortality, survival with dependence on RRT, ICU stay, hospital length of stay (HLOS). References were first independently examined at abstract level by three investigators, with divergences resolved by consensus. If potentially pertinent, articles were retrieved as full articles. Two investigators independently assessed compliance to selection criteria and selected studies for the final analysis, with divergences resolved by consensus.

### Data abstraction and study

Baseline and outcome data were independently abstracted by three investigators (Tables 1 and 2). At least two separate attempts at contacting original authors were made in cases of missing data. The co-primary endpoints of the present review were mortality at the longest follow-up available and survival with dependence on RRT. Secondary endpoints were ICU stay (days) and HLOS (days). Adverse effects (bleeding complications, blood transfusions, catheter-related bacteremia, rhythm disturbances, thrombocytopenia) were also analysed. Subanalysis were performed on the subgroup of patients who underwent cardiac surgery and on general ICU patients.

**Table 1:** Study characteristics

Author	Yournal	Year	Setting	Number of randomized patients	Early RRT criteria	Late RRT criteria
Barbar SD	NEJM	2018	ICU	488	< 12 h	Hyper-kalemia (potassium level > 6.5 mmol per liter), metabolic acidosis (pH < 7.15), or fluid overload (extravascular fluid overload that was refractory to diuretics, with pulmonary edema).
Bouman CSC	Crit Care Med.	2002	ICU	106	< 12 h	> 12 h
Combes A	Am J Respir Crit Care Med.	2015	Post–cardiac surgery shock	224	< 24 h and continued at least 48 h	Creatinine > 4 mg/dL; Preoperative creatinine × 3 or Urine output < 0.3 ml/kg/h /24 h or Urea > 36 mmol/L or Life-threatening hyperkalemia
Durmaz I	Ann Thorac Surg	2003	Patients undergoing CABG	44	Postoperative creatinine > 10% within 48 h	Postoperative creatinine > 50% or diuresis < 400 ml/24 h and K+/H+ unresponsive to therapy
Gaudry S	NEJM	2016	ICU	619	< 6 h stage 3 AKI	Oliguria or anuria for more than 72 h after randomization; Blood urea nitrogen of more than 112 md/dl (40 mmol/liter); Serum potassium concentration of more than 6 mmol/liter or more than 5.5 mmol/liter despite medical treatment; pH below 7.15 in a context of pure metabolic acidosis (PaCO2 < 35 mmHg) or in a context of mixed acidosis with PaCO2 of 50 mmHg or more without possibility of increasing alveolar ventilation; Acute pulmonary edema due to fluid overload leading to severe hypoxemia requiring oxygen flow rate of more than 5 l/min to maintain SpO2 of more than 95% or requiring an FiO2 greater than 50% in patients already on invasive or non-invasive mechanical ventilation and despite diuretic therapy
Jamale TE	Am J Kidney Dis.	2013	Patients With Community-Acquired AKI	208	Creatinine level > 618 μmol/L	Treatment-refractory hyperkalemia,volume overload, and acidosis.,uremic nausea and anorexia leading to inability to maintain nutrient intake
Payen D	Crit Care Med.	2009	ICU	76	Protocolized RRT for 96 h at the diagnosis of ‘sepsis’. Mean time to initiation of RRT not specified	Standard sepsis management
Sugahara S	Hemodial int	2004	Coronary artery bypass surgery.	28	diuresis < 30 ml/hr. for 3 h or < 750 ml/day	diuresis < 20 ml/hr. for 2 h or < 500 ml/day
Wald R	Kidney Int.	2015	ICU	100	< 12 h	Volume overload and/or oligoanuria; PaO2/FiO2 o200, serum potassium concentration 6 mmol/l
Zarbock A	JAMA	2016	ICU	231	< 8 h diagnosis of stage 2 AKI	within 12 h of stage 3 AKI

**Table 2:** Primary and secondary outcomes, adverse events and sensitivity analyses

Outcome	Number of included trials	Early RRT patients	Control patients	OR or MD	95% CI	P for effect	P for heterogeneity	I^2^ (%)
Overall trials	10	1073	1141					
*Primary outcomes*								
-Mortality	10	1073	1141	0.99	0.66 to 1.50	0.97	< 0.0001	74
General ICU patients	7	926	992	1.15	0.79 to 1.68	0.47	0.005	68
Cardiac surgery patients	3	147	149	0.19	0.01 to 2.66	0.22	0.003	88
SENSITIVITY ANALYSIS (including only low risk of bias studies)	4	771	865	1.13	0.66 to 1.95	0.65	0.0005	83
-Survival with dependence on RRT	6	931	939	0.86	0.54 to 1.37	0.51	0.54	0
General ICU patients	5	819	827	0.86	0.54 to 1.37	0.51	0.54	0
Cardiac surgery patients	1	112	112	na	na	na	na	na
SENSITIVITY ANALYSIS (including only low risk of bias studies)	4	771	775	0.90	0.56 to 1.45	0.66	0.53	0
SENSITIVITY ANALYSIS (removing 1 study at time)	All 95% CIs of OR > 1 and *p* < 0.05							
*Secondary outcomes*								
-ICU stay (days)	6	808	780	−0.87	−2.02 to 0.27	0.14	0.11	44
-HLOS (days)	6	808	780	−2.92	−4.47 to − 1.38	0.0002	0.35	10
SENSITIVITY ANALYSIS (including only low risk of bias studies)	3	669	669	−3.03	−5.36 to −0.71	0.10	0.11	58
*Adverse events*								
-Bleeding Complications	8	1038	1014	0.90	0.70 to 1.17	0.44	0.68	0
-Blood transfusions	3	659	656	0.94	0.72 to 1.23	0.65	0.62	0
-Catheter-related bacteremia	4	498	505	1.70	0.98 to 2.93	0.06	0.53	0
-Rhythm disturbances	6	775	783	1.09	0.56 to 2.13	0.80	0.09	47
-Thrombocytopenia	2	423	420	1.42	0.76 to 2.63	0.27	0.05	75

*RRT* renal replacement therapy, *OR* relative risk, *MD* mean difference, *CI* confidence interval, *P p*-value, *ICU* intensive care unit, *HLOS* hospital length of stay

The internal validity and risk of bias of included trials was appraised by two independent reviewers according to the latest version of the “Risk of bias assessment tool” developed by The Cochrane collaboration [13]. Publication bias was assessed by visually inspecting funnel plots. Sensitivity analyses were performed by sequentially removing each study and reanalyzing the remaining dataset (producing a new analysis for each study removed) and by analyzing only data from studies with low risk of bias.

### Data analysis and synthesis

Computations were performed with Review Manager version 5.2. Hypothesis of statistical heterogeneity was tested by means of Cochran Q test, with statistical significance set at the two-tailed 0.10 level, whereas extent of statistical consistency was measured with I^2^, defined as 100% X (Q-df)/Q, where Q is Cochran’s heterogeneity statistic and df the degrees of freedom. Binary outcomes from individual studies were analysed to compute individual and pooled odds ratio (OR) with pertinent 95% confidence interval (CI), by means of Mantel-Haenszel method and with a fixed-effect model in case of low statistical inconsistency (I^2^ < 25%) or with random-effect model (which better accommodates clinical and statistical variations) in case of moderate or high statistical inconsistency (I^2^ > 25%). To evaluate if the small study effect will have an influence on the treatment effect estimate, in case of evidence of between-study heterogeneity (I^2^ > 25), we compared the results of both fixed and random effect models. Sensitivity analyses were performed by sequentially removing each study and reanalysing the remaining dataset (producing a new analysis for each study removed) and by analysing only data from studies with low risk of bias. Statistical significance was set at the two tailed 0.05 level for hypothesis testing. Unadjusted *p* values are reported throughout. This study was performed in compliance with The Cochrane Collaboration and Preferred Reporting Items for Systematic Reviews and Meta-Analyses guidelines [13–15].

## Results

### Study characteristics

**Fig. 1:**
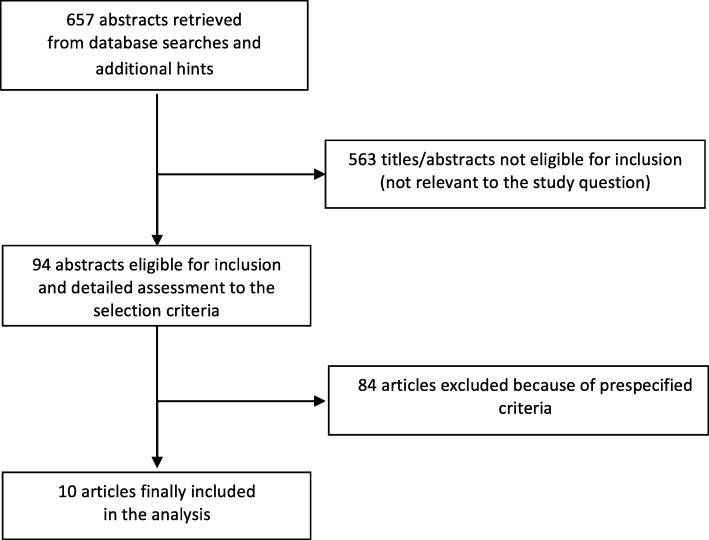
Flow-chart for study selection

Database searches, snowballing, and contacts with experts yielded a total of 657 articles (updated October 15th 2018). The flow chart to select the final 10 manuscripts trials [12, 16–24] is detailed in Fig. 1. Excluding 563 non-pertinent titles or abstracts, we retrieved in complete form and assessed 94 studies according to the selection criteria. 84 studies were further excluded because of our prespecified exclusion criteria. (Fig. 1).

The 10 included trials randomized 2214 patients, 1073 to earlier initiation of RRT and 1141 to later initiation. (Table 1) Clinical heterogeneity was mostly due to setting and criteria for early and late initiation of RRT. (Table 1) Indeed three trials were performed in patients who underwent cardiac surgery [19, 20, 23] while the other seven were performed in general ICU patients [12, 16–18, 21, 22, 24] (Table 1).

### Quantitative data synthesis

**Fig. 2:**
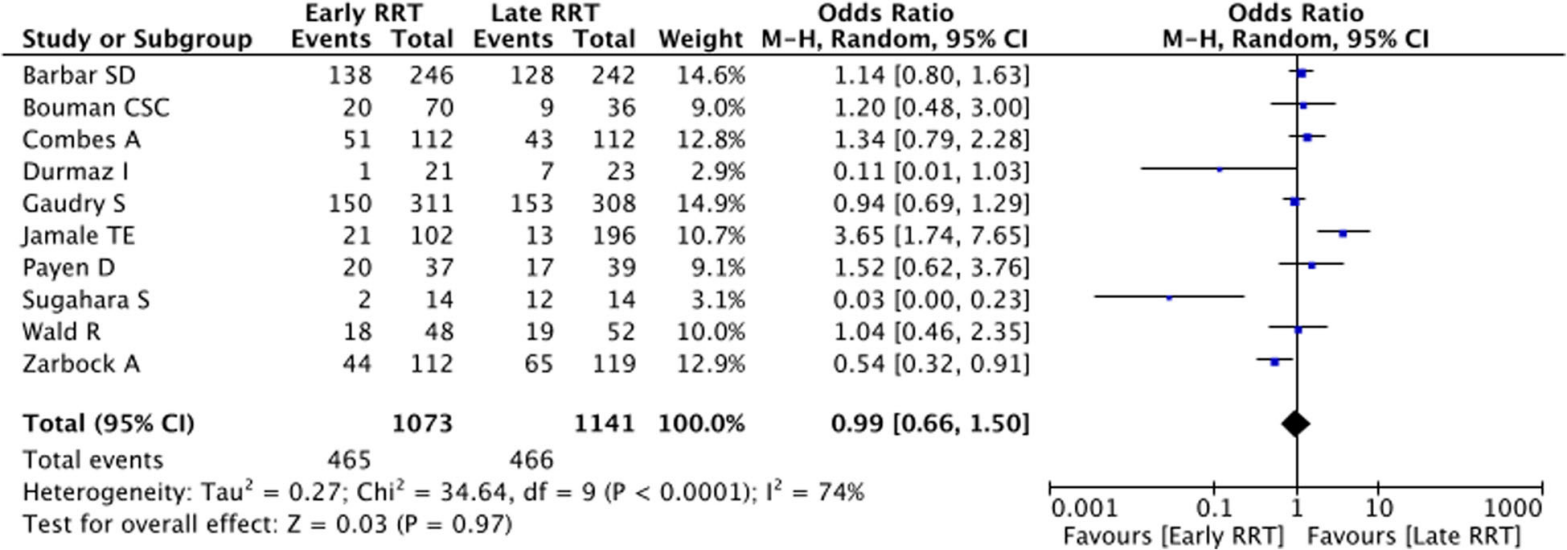
Forest plot for mortality

**Fig. 3:**
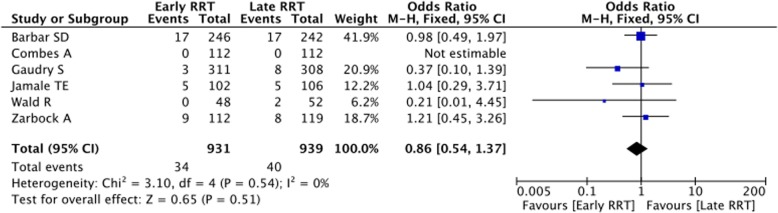
Forest plot for survival with dependence on RRT

Overall analysis showed that early initiation of RRT does not improve outcome of critically ill patients with AKI. In fact, no differences in mortality (Fig. 2) and survival with dependence on RRT were observed between groups. (Fig. 3) Results were confirmed at sensitivity analyses and the funnel plot illustrated in the Additional file 1. (Table 2; Additional file 1: Figures S1 and S2).

On the contrary, early initiation of RRT was associated with a significant reduction in HLOS. (Additional file 1: Figures S3 and S4) Nonetheless results were not confirmed at sensitivity analyses. (Table 2) Visual inspection of funnel plots did not identify a skewed or asymmetrical shape for the primary endpoints (Additional file 1: Figures S5 and S6).

No differences in occurrence of adverse events were observed (Table 2).

## Discussion

Our meta-analyses suggests that early initiation of RRT does not improve clinically relevant outcomes of critically ill patients with AKI. In fact, mortality and survival with dependence on RRT don’t differ between patients who received early RRT and patients who received standard treatment. Moreover, although we found an overall significant reduction in HLOS in the subgroup of patients who received early RRT, these positive results were not confirmed in the high-quality studies. In addition, we didn’t find a subgroup of patients in which early initiation of RRT could me more beneficial since outcome did not improve both in cardiac surgery patients and general ICU patients.

Our results diverge from the results of recent meta-analyses on this topic, while confirm the results of the less recent meta-analyses performed by Wierstra et al. [9–11]. Nonetheless, the conclusions of Wiestra at al. were weaker since were based on fewer, lower quality studies and didn’t include the most recent, high-quality trials published in the last year. We updated their results with three recently published manuscripts, [12, 16, 17] thus increasing the number of patients by more than 100% (up to 2214 overall randomized patients included in our meta-analysis) and allowing to have more robust data. Our results are consistent with the results of another recent meta-analyses performed by Feng et al. [25]. Nonetheless, Feng et al. limited their analyses to mortality and ICU and hospital length of stay, without considering adverse effects. Moreover they did not perform any subanalyses on general ICU patients or cardiac surgery patients, therefore drawing weaker conclusions. On the contrary, Moreira et al. found an increased risk of catheter-related bloodstream infection when renal replacement therapy was initiated early. Moreira FT, et al. [10] our study doesn’t confirm this finding.

Although our meta-analysis includes all the randomized clinical trials ever published on early vs late RRT and two large, recent, high-quality RCTs, the optimal timing of initiating RRT remains unclear. Actually, we couldn’t add great new findings to previous published meta-analyses. A reasonable explanation for this is that our study is still underpowered for mortality. Moreover, the analyzed studies were conducted over a wide range of time, during which the management of AKI patients has greately changed. In fact, in the last decade the Kidney Disease Improving Global Outcomes (KDIGO) Clinical Practice Guideline contributed to standardize AKI treatment. This means that the more recent studies published after 2010 failed to show a significant survival benefit from early RRT treatment, while a reduction in mortality was shown by older studies. Initiation of RRT, to some extent, depends on creatinine level and urine output, namely, the KDIGO criteria. Therefore, one of the main limitations of our meta-analysis and of all the performed and ongoing trials is the lack of definition of “early” versus “late” criteria, that varied among the included studies and may have led to great differences in the requirements for RRT and their therapeutic impact. Larger, well conducted RCTs should be performed to further clarify this issue. Actually, there is another ongoing RCT that will probably provide additional informations on the optimal timing of starting RRT in critically ill patients admitted to general ICU (STARRT-AKI, NCT02568722). Unfortunately, given the previous reported limitations, this trial will not probably allow to draw definitive conclusions on the optimal timing of starting RRT in critically ill patients.

## Conclusions

Our meta-analysis supports the notion that early initiation of RRT in critically ill patients with AKI does not provide a clinically relevant advantage when compared with standard/late initiation. Large, multicenter RCTs are warranted to clarify the optimal timing of starting RRT. Based on the limitations of the data available for our analysis, future work in the following areas is desirable: (1) stardardized definition of “early” and “late” initiation of RRT; (2) special populations such as the septic shock patients or post cardiac surgery patients; (3) an assessment of the performance of the different RRT modalities and dosage options.

## Additional file

Additional file 1: Supplemental material: Pubmed search strategy, additional figures (Funnel plots and forest plots); Study quality appraisal. (DOCX 156 kb)

## Abbreviations

AKI: Acute kidney injury
HLOS: Hospital length of stay
HRQoL: Health-related quality of life
ICU: Intensive care unit
KDIGO: Kidney Disease Improving Global Outcomes (KDIGO)
RCT: Randomized clinical trials
RRT: Renal replacement therapy
