# Supplementary material for: Early initiation of renal replacement therapy in critically ill patients: a meta-analysis of randomized clinical trials
Source: BMC Anesthesiol. 2019 May 1;19:62. doi: 10.1186/s12871-019-0733-7 (PMC6492439; doi:10.1186/s12871-019-0733-7)
Supplement: Supplementary file 1 — Supplemental material: Pubmed search strategy, additional figures (Funnel plots and forest plots); Study quality appraisal. (DOCX 156 kb) [file 12871_2019_733_MOESM1_ESM.docx]

**Additional file**

**PUBMED SEARCH STRATEGY**

Renal replacement therapy critical ill AND (randomized controlled trial[pt] OR controlled clinical trial[pt] OR randomized controlled trials[mh] OR random allocation[mh] OR double-blind method[mh] OR single-blind method[mh] OR clinical trial[pt] OR clinical trials[mh] OR (clinical trial[tw] OR ((singl*[tw] OR doubl*[tw] OR trebl*[tw] OR tripl*[tw]) AND (mask*[tw] OR blind[tw])) OR (latin square[tw]) OR placebos[mh] OR placebo*[tw] OR random*[tw] OR research design[mh:noexp] OR follow-up studies[mh] OR prospective studies[mh] OR cross-over studies[mh] OR control*[tw] OR prospectiv*[tw] OR volunteer*[tw]) NOT (animal[mh] NOT human[mh]) NOT (comment[pt] OR editorial[pt] OR meta-analysis[pt] OR practice-guideline[pt] OR review[pt]))

**Figure S1: Funnel plot for mortality**

**
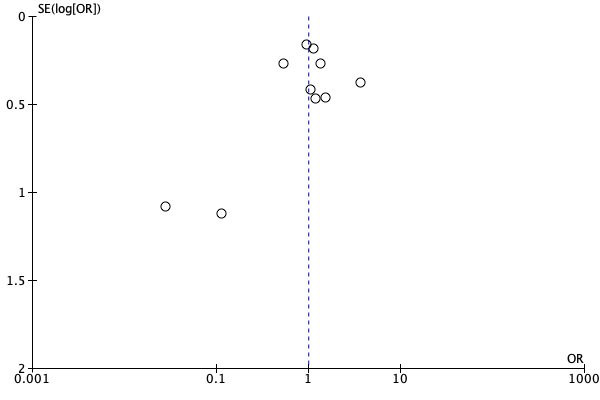
**

**Figure S2: Funnel plot for survival with dependence on RRT**

**
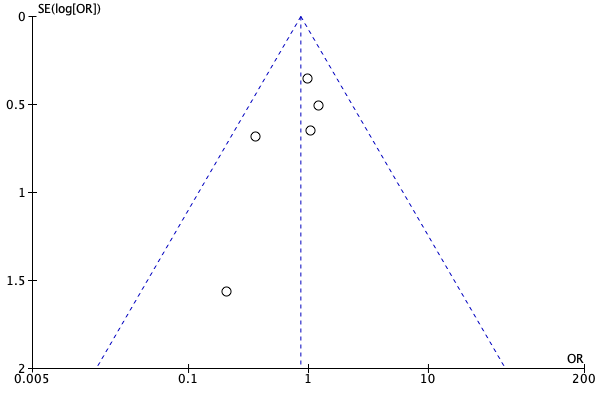
**

**Figure S3: Forest plot for ICU stay**

**
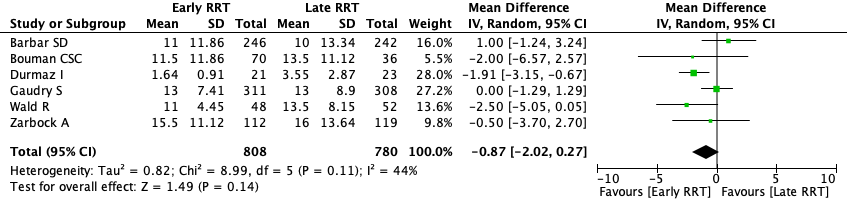
**

**Figure S4: : Forest plot for HLOS**

**
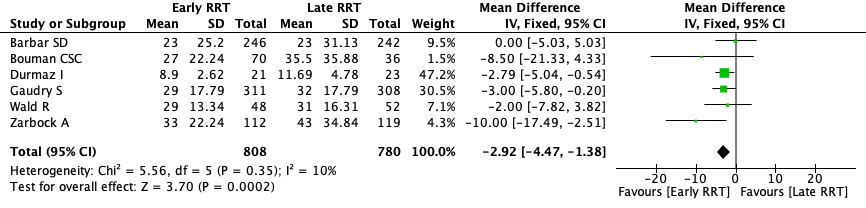
**

**Figure S5: Funnel plot for ICU stay**

**
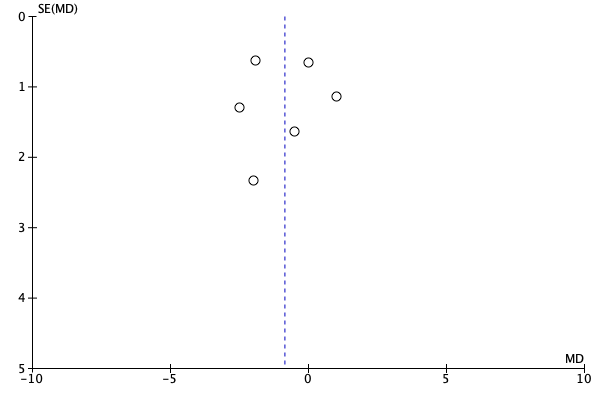
**

**Figure S6: Funnel plot for HLOS**

**
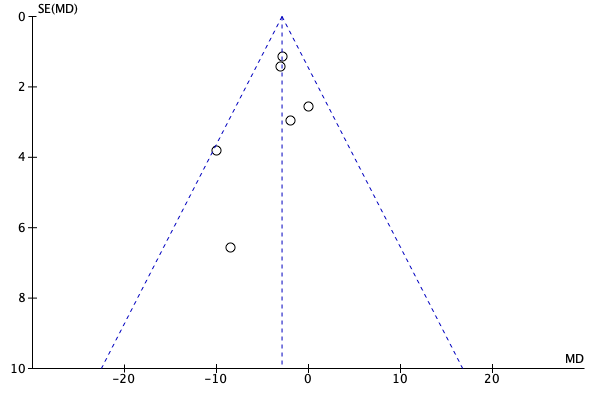
**

**Study quality appraisal**

| **Trial** | **Entry** | **Judgment** | **Value** | **Description** |
| --- | --- | --- | --- | --- |
| Barbar SD | Adequate sequence generation? | Yes | 1 |  |
|  | Allocation concealment? | Yes | 1 |  |
|  | Blinding of participants and personnel? | No | 0 |  |
|  | Blinding of outcome assessment? | No | 0 |  |
|  | Complete outcome data addressed? | Yes | 1 |  |
|  | Free of selective reporting? | Yes | 1 |  |
|  | Free of other bias? | Yes | 1 |  |
|  | OVERALL RISK OF BIAS | Low | 5 |  |
| Bouman CSC | Adequate sequence generation? | Unclear | 0,5 |  |
|  | Allocation concealment? | No | 0 |  |
|  | Blinding of participants and personnel? | No | 0 |  |
|  | Blinding of outcome assessment? | No | 0 |  |
|  | Complete outcome data addressed? | Yes | 1 |  |
|  | Free of selective reporting? | Yes | 1 |  |
|  | Free of other bias? | Yes | 1 |  |
|  | OVERALL RISK OF BIAS | Moderate | 3,5 |  |
| Combes A | Adequate sequence generation? | Yes | 1 |  |
|  | Allocation concealment? | No | 0 |  |
|  | Blinding of participants and personnel? | No | 0 |  |
|  | Blinding of outcome assessment? | No | 0 |  |
|  | Complete outcome data addressed? | Yes | 1 |  |
|  | Free of selective reporting? | Yes | 1 |  |
|  | Free of other bias? | Yes | 1 |  |
|  | OVERALL RISK OF BIAS | Moderate | 4 |  |
| Durmaz I | Adequate sequence generation? | No | 0 |  |
|  | Allocation concealment? | No | 0 |  |
|  | Blinding of participants and personnel? | No | 0 |  |
|  | Blinding of outcome assessment? | No | 0 |  |
|  | Complete outcome data addressed? | Yes | 1 |  |
|  | Free of selective reporting? | Yes | 1 |  |
|  | Free of other bias? | Yes | 1 |  |
|  | OVERALL RISK OF BIAS | High | 3 |  |
| Gaudry S | Adequate sequence generation? | Yes | 1 |  |
|  | Allocation concealment? | Yes | 1 |  |
|  | Blinding of participants and personnel? | No | 0 |  |
|  | Blinding of outcome assessment? | No | 0 |  |
|  | Complete outcome data addressed? | Yes | 1 |  |
|  | Free of selective reporting? | Yes | 1 |  |
|  | Free of other bias? | Yes | 1 |  |
|  | OVERALL RISK OF BIAS | Low | 5 |  |
| Jamale TE | Adequate sequence generation? | Yes | 1 |  |
|  | Allocation concealment? | Yes | 1 |  |
|  | Blinding of participants and personnel? | No | 0 |  |
|  | Blinding of outcome assessment? | No | 0 |  |
|  | Complete outcome data addressed? | Yes | 1 |  |
|  | Free of selective reporting? | Yes | 1 |  |
|  | Free of other bias? | Yes | 1 |  |
|  | OVERALL RISK OF BIAS | Low | 5 |  |
| Payen D | Adequate sequence generation? | Unclear | 0,5 |  |
|  | Allocation concealment? | No | 0 |  |
|  | Blinding of participants and personnel? | No | 0 |  |
|  | Blinding of outcome assessment? | No | 0 |  |
|  | Complete outcome data addressed? | Yes | 1 |  |
|  | Free of selective reporting? | Yes | 1 |  |
|  | Free of other bias? | Yes | 1 |  |
|  | OVERALL RISK OF BIAS | Moderate | 3,5 |  |
| Sugahara S | Adequate sequence generation? | Unclear | 0,5 |  |
|  | Allocation concealment? | No | 0 |  |
|  | Blinding of participants and personnel? | No | 0 |  |
|  | Blinding of outcome assessment? | No | 0 |  |
|  | Complete outcome data addressed? | Yes | 1 |  |
|  | Free of selective reporting? | Yes | 1 |  |
|  | Free of other bias? | Yes | 1 |  |
|  | OVERALL RISK OF BIAS | Moderate | 3,5 |  |
| Wald R | Adequate sequence generation? | Yes | 1 |  |
|  | Allocation concealment? | Unclear | 0,5 |  |
|  | Blinding of participants and personnel? | No | 0 |  |
|  | Blinding of outcome assessment? | No | 0 |  |
|  | Complete outcome data addressed? | Yes | 1 |  |
|  | Free of selective reporting? | Yes | 1 |  |
|  | Free of other bias? | Yes | 1 |  |
|  | OVERALL RISK OF BIAS | Moderate | 4,5 |  |
| Zarbock A | Adequate sequence generation? | Yes | 1 |  |
|  | Allocation concealment? | Yes | 1 |  |
|  | Blinding of participants and personnel? | No | 0 |  |
|  | Blinding of outcome assessment? | No | 0 |  |
|  | Complete outcome data addressed? | Yes | 1 |  |
|  | Free of selective reporting? | Yes | 1 |  |
|  | Free of other bias? | Yes | 1 |  |
|  | OVERALL RISK OF BIAS | Low | 5 |  |

Yes=1; Unclear=0.5; No=0; ≥5Low; >3-<5Moderate; ≤3 High
